# Distinct effects of empathy on self–other processing revealed by different behavioral and EEG indices

**DOI:** 10.3758/s13415-026-01416-2

**Published:** 2026-03-04

**Authors:** Shih-Yu Lo, Jen-Chung Wang, Kuan-Chih Huang, Chih-Mao Huang, Chin-Teng Lin

**Affiliations:** 1 Institute of Communication Studies, National Yang Ming Chiao Tung University, Hsinchu, Taiwan; 2 Institute of Molecular Medicine and Bioengineering, National Yang Ming Chiao Tung University, Hsinchu, Taiwan; 3 Institute of Electrical Control Engineering, National Yang Ming Chiao Tung University, Hsinchu, Taiwan; 4 Brain Science and Technology Center, College of Electrical and Computer Engineering, National Yang Ming Chiao Tung University, Hsinchu, Taiwan; 5 Department of Psychology, The University of Hong Kong, Hong Kong, China; 6 Australian AI Institute, School of Computer Science, FEIT, University of Technology Sydney, Sydney, Australia

**Keywords:** Empathy, Event-related potentials, Self-representation, P3, Late positive component

## Abstract

The self-prioritization effect refers to the tendency for individuals to perceive self-related stimuli more accurately than other-related stimuli. We leveraged this phenomenon to investigate the mechanisms underlying empathy. According to the self–other merging hypothesis, empathy broadens self-representation to encompass others, predicting a reduced self-prioritization effect. In contrast, the self–other distinction hypothesis posits that empathy heightens the differentiation between self and other, predicting an increased self-prioritization effect. To test these competing hypotheses, we employed a perceptual task combined with electroencephalogram (EEG) recordings, and analyzed the data by using signal detection theory. Fifty-two participants aged 19 to 33 years were randomly assigned to either a high-empathy or low-empathy group. Participants listened to an interview and were instructed to either empathize with or remain neutral toward the interviewee. Subsequently, they completed a shape–label matching task using geometrical shapes associated with the self, the interviewer, and the interviewee. The self-prioritization effect was measured by performance differences between self-related and other-related stimuli. Different performance indices provided support for each hypothesis. Specifically, the patterns observed in the sensitivity index (*d’*) and the lateral positive component (LPC) of the event-related potential supported the self–other distinction hypothesis at the perceptual and attentional levels. In contrast, the patterns in the response bias index *(c)* and reaction time supported the self–other merging hypothesis at the response level. These findings suggest that empathy influences self-representation in distinct ways across different levels of processing.

**Supplementary Information:**

The online version contains supplementary material available at 10.3758/s13415-026-01416-2.

## Introduction

From an evolutionary standpoint, self-representation is crucial for survival, because it enables individuals to identify their own boundaries to protect themselves from potential threats. Its significance is supported by developmental studies, suggesting that self-representation may emerge as early as the first 2 years of life (Lewis & Carmody, 2008).

However, the boundaries of the self are not rigid. Under certain conditions, they can expand to include external objects or individuals. A classic illustration of this phenomenon is the rubber-hand illusion (Ehrsson et al., 2004), in which synchronized stroking of both the participant’s hand (out of sight) and a visible fake rubber hand leads the participants to experience the rubber hand as an extension of their own body. This phenomenon exemplifies the malleability of self-representation.

### Empathy and self–other merging

This expansion of self-representation may be connected to empathy, a multifaceted concept involving sharing and experiencing the feelings of others (Decety & Lamm, 2006). Empathy is commonly divided into cognitive and affective components (Rogers et al., 2007). Cognitive empathy focuses on the process of understanding another person’s perspective, while affective empathy involves sharing or mirroring the emotional states of others. Empathy can also be classified as dispositional empathy, a personality trait (Davis, 1983), or situational empathy, influenced by external conditions (Batson et al., 1997; Lo, 2021; Lo et al., 2022).

Batson et al. (1997) provided foundational evidence for empathy-induced self–other merging. Participants who were instructed to empathize with a fictional character reported higher ratings of self–other overlap using the Inclusion of Others scale (Aron et al., 1992). Additional support comes from studies showing that trait empathy is correlated with susceptibility to body illusions (Seiryte & Rusconi, 2015) and from research on mirror-touch synesthetes (individuals who experience tactile sensations when observing others being touched), who also report elevated empathy levels (Banissy & Ward, 2007).

Neuroimaging studies further support self–other merging. Wu et al. (2010) found that Tibetan participants, influenced by Buddhist teachings that emphasize reduced attachment to the self, showed more similar neural responses to self- and other-related information compared with non-Tibetan participants. Similarly, Riečanský et al. (2020) found that participants with stronger empathy-related neural activations also reported higher perceived bodily self-attribution (e.g., feeling another person’s body part as part of one’s own). Chen et al. (2020) showed that individuals with interdependent self-construal, who view the self as inherently connected to others, exhibited stronger neural responses to others’ pain.

### Empathy and self–other distinction

In contrast, some researchers argue that empathy requires a clear differentiation between self and others (Decety & Lamm, 2006; Krol & Bartz, 2022). In the self-to-other model of empathy proposed by Bird and Viding (2014), the process of sharing another person’s affective state is referred to as “emotional contagion.” However, emotional contagion alone does not constitute empathy. Rather, one must explicitly recognize that the experienced affect originates from another person, an act of “self–other tagging” that is critical for genuine empathic engagement.

Empirical support for self–other distinction can be seen in the study of Mattan et al. (2016). In their study, participants were presented with two avatars (digital representation): one labeled as “self” and the other as “other.” Their task was to report what each avatar observed in a simulated environment. Performance was better when both avatars held the same perspective than when they held different perspectives, indicating a congruency effect. Importantly, this effect was negatively correlated with dispositional empathy; individuals with higher empathy were less disrupted by incongruent other-related information, presumably because they maintained a clearer boundary between self and other.

Further evidence comes from De Guzman et al. (2016), who found that training participants in self–other control during motor tasks enhanced both their corticospinal empathic responses (Experiment 1) and self-reported empathy (Experiment 2). Chiu and Yeh (2018) showed that the ability to shift perspectives (from another’s back to one’s own) was positively associated with emotional empathy. Likewise, Little et al. (2023) demonstrated that individuals with high trait empathy were more adept at distinguishing between their own and others’ mental states.

### Self-other control

Taken together, these studies reveal a paradox in how empathy relates to self-representation. Empathy can heighten individuals’ awareness to the distinction between self and others; conversely, it blurs the boundaries between self and others. The dual role of empathy has been recognized in previous studies. Bird and Viding (2014) described them as emotional contagion and self–other switch, whereas Lamm et al. (2016) refer to them as affective sharing and self–other distinction*.* A more neutral term, self–other control, has been proposed to capture the coordination of these seemingly opposing mechanisms (Brass & Heyes, 2005; Decety & Sommerville, 2003; Sowden & Shah, 2014; Spengler et al., 2009). Emotional contagion promotes self–other merging, yet empathy-related tasks also require individuals to determine whether an emotion arises from themselves or from another person. Consistent with this, Santiesteban et al. (2012) demonstrated that training individuals to inhibit their tendency to imitate improved their performance on a visual perspective-taking task, suggesting that self-other control supports successful empathetic processing.

To empirically investigate how empathy influences these components of self-other control, an objective and sensitive measure of self-representation is required. The shape–label matching task developed by Sui et al. (2012) offers an objective measure of self-representation. In this task, participants associate geometrical shapes (e.g., a circle, a triangle, and a square) with social labels (e.g., self, friend, and stranger). During the main task, they judge whether presented shape–label pairs match the previously assigned associations. Sui et al. (2012) observed a self-prioritization effect, where participants responded more accurately to patterns associated with the self than to those associated with a friend or stranger. Thus, the extent of the self-prioritization effect could be an indicator of self-representation. If the function of empathy is to facilitate self–other merging, higher levels of empathy should diminish the self-prioritization effect. Conversely, if the function of empathy is to foster self–other distinction, heightened empathy should amplify the self-prioritization effect.

### Empathy effect at multiple levels of self-representation

The task described above offers a valuable tool measuring self-representation. To investigate whether empathy involves self–other merging or self–other distinction, one potential approach is to induce varying levels of empathy and observe changes in the effect of self-prioritization. This was the focus of the current study.

Human cognitive processing can be viewed as a series of interconnected stages that work together to support our ability to perceive and interact with the world. Therefore, it is possible that empathy exerts its effects differently across these stages. To gain a deeper insight into how empathy influences self-representation in different levels of processing, we analyzed the behavioral data with the theoretical framework of the signal detection theory (SDT), as in Sui et al. (2012) and Lo (2021). The SDT allows for the estimation of two key indices: the index of perceptual sensitivity, or *d’*, and the index of response bias, or *c* (Stanislaw & Todorov, 1999).

In addition, we recorded and analyzed event-related potentials (ERPs), focusing on the N1, P3, and late positive component (LPC). The N1 component appears approximately 140–190 ms and has been associated with visual attention (Hillyard et al., 1998). P3, also known as P300, is an ERP component that typically appears approximately 250–500 ms after a target event, which is also associated with attentional processing (Donchin & Coles, 1988; Johnson, 1988). Recent research has revealed the links between the N1 and P3 components and the neural representations of the self, with greater N1 (Amodeo et al., 2024; Sui et al., 2023) and P3 amplitudes (Gray et al., 2004; Liu et al., 2019; Niu et al., 2020; Tacikowski & Nowicka, 2010; Xia et al., 2018) observed for self-related stimuli compared to stimuli unrelated to the self.

Another component closely related to P3 is the late positive component (LPC), a parietally distributed positive waveform observed between approximately 400–800 ms. The LPC is frequently reported in memory studies, where previously encountered (“old”) stimuli elicit larger LPC amplitudes than novel (“new”) stimuli in recognition tasks (Friedman & Johnson, 2000). Due to its overlapping time window with P3, LPC is sometimes considered as a broader and more nebulous term encompassing P3 (or P300) (Polich, 2007). Some studies even refer to P3 as a subcomponent of a broader category of LPC (Barry et al., 2020; Kissler et al., 2009). Importantly, LPC amplitude is also enhanced for self-related information. For example, Rubianes et al. (2021) found a larger LPC (450–600 ms) in response to one’s own face compared with a close friend’s face. This self-prioritization effect extended to comparisons between the past-self and the past-close-friend, highlighting LPC’s involvement in representing abstract, temporally invariant self-representations. Tacikowski and Nowicka (2010) found that self-related names and photos elicited enhanced amplitudes in the 350–850-ms time window, which spans the range typically associated with both P3 and LPC components.

Recently, self-prioritization effect in the ERPs have also been reported in the shape–label matching paradigm. Sui et al. (2023) observed self-bias effects in the N1 (time window: 147–167 ms) and P3 (time window: 340–500 ms) components. In another study using the same task, Amodeo et al. (2024) found comparable self-prioritization in the N1 (190–280 ms) for both autistic and nonautistic participants. However, the autistic group showed reduced self-bias in the P3 component (500–700 ms). Given the relatively late latency of the P3 effect in that study, we refer to it using the more general term *late positive component (LPC)* for clarity.

### Overview of the present study

This study examines how empathy influences self–other processing; specifically, whether it promotes self–other merging or self–other distinction. Empathy was manipulated using the procedure of Batson et al. (1997), in which participants were instructed either to empathize with a person experiencing emotional distress or to remain emotionally neutral.

Following the empathy manipulation, participants completed a shape–label matching task, matching geometrical shapes to labels representing themselves and two other persons. If empathy induces self–other merging, participants in the high-empathy group should show a reduced self-prioritization effect. If it instead enhances self–other distinction, they should show an increased self-prioritization effect.

Because merging and distinction may occur at different stages of processing, we analyzed the data using SDT. The perceptual index (*d’)* and response index (*c)* were used to isolate effects at the perceptual versus response stages. Reaction times were also recorded, following prior work using the shape-label matching paradigm (Sui et al., 2012, 2023), although this measure captures overall processing rather than specific stages. To further examine temporal dynamics, EEG data were collected (Huang et al., 2021). The N1, P3, and LPC components were analyzed to characterize how empathy modulates attentional processing across different time windows.

## Methods

### Participants

We conducted a power analysis based on the study of Little et al. (2023). In their study, the effect size *η*^*2*^_*p*_ of empathy on self–other discrimination was 0.137. Based on this effect size, and the calculation result of G*Power 3 (Faul et al., 2007), a sample size of 52 was required to achieve a power of 0.8. Thus, we recruited 52 participants (26 females; mean age: 22.15 years; age range: 19–33 years). All the participants had normal or corrected-to-normal vision, and none of them reported a history of psychological or neurological disorders.

### Procedure

After obtaining informed consent, we conducted the electroencephalogram (EEG) setup procedure. EEG signals were recorded using a 32-electrode cap (Ag/AgCl), referenced to the average of the mastoids (M1 and M2), following the International 10–20 system, and amplified using the Syn-Amp2 amplifier (Compumedics Neuroscan Inc., Australia). Conductive gel was applied to maintain electrode impedance below 5 kΩ at each electrode.

Empathy was manipulated in the same manner as in Batson et al. (1997). Participants were randomly assigned either to the high-empathy group or low-empathy group. Those in the high-empathy group received the following instructions:

"You will be listening to an interview shortly. During the interview, please try your best to imagine the interviewee's feelings and consider the impact of this incident on them or others. Please pay attention to the details in the interview, and then try your best to imagine the interviewee's feelings or the impact of his or her behavior on others."

Meanwhile, participants in the low-empathy group were instructed as follows:

"You will be listening to an interview shortly. During the interview, please look at the interviewee's experience as objectively as possible, and try to distance yourself from the situation. To be as objective as possible, please refrain from imagining the interviewee's experiences, feelings, or the impact of their actions on others."

Participants then listened to an audio-recorded interview between a program host and a university student named Lin, who had recently lost his (her) parents in a tragic accident. To care for two younger siblings, Lin had to give up his (her) dream of becoming a pilot and took several part-time jobs to support the family (see Supplementary Information: Appendix A). Lin’s voice was portrayed by either a male or a female actor, matched to each participant’s gender.

Following the interview, the participant completed the experimental task. Seated approximately 50 cm in front of a 24-inch monitor (BenQ ZOWIE XL2430, resolution: 1920 × 1080), they were first shown a task-overview display explaining how to perform the task. One-third of the participants were instructed to associate “self” with a circle, “Lin” with a square, and “host” with a triangle; another third of participants were instructed to associate “self” with a square, “Lin” with a triangle, and “host” with a circle; and the remaining third of participants were instructed to associate “self” with a triangle, “Lin” with a circle, and “host” with a square. The labels of “Lin” and “host” corresponded to the interviewee and the program host.

**Fig. 1:**
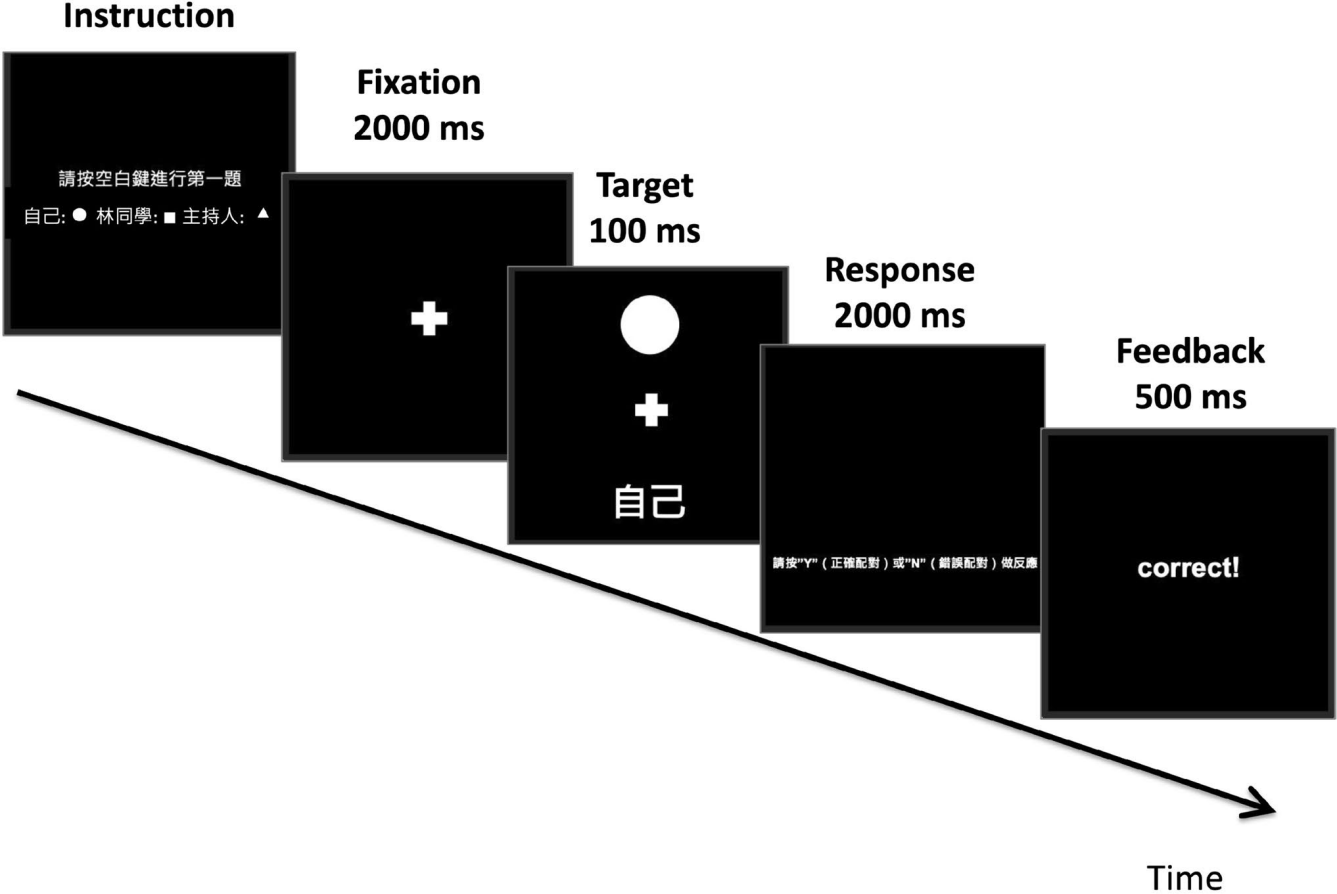
The stimulus sequence of the experimental task. The images are only for demonstration purposes and are not proportionally scaled. The text in the instruction display is translated as “please press the space bar to proceed to Trial 1; self: ● Lin: ■ host: ▴,” the text in the target display is translated as “self,” and the text in the response display is translated as “please make a response by pressing y (correct match) or n (incorrect match).”

The task was programmed by using Presentation 22. At the start of each trial (Fig. 1), an instruction display informed the participants of the association rule and the number of the current trial. Once ready, they pressed the spacebar to proceed. A fixation cross (each arm of 100 × 10 pixels) was shown for 2,000 ms, followed by the target display. The target display included the fixation cross along with a geometrical shape and a social label. The shape could be a triangle (side length: 200 pixels), a square (side length: 150 pixels), or a circle (diameter: 200 pixels), centered 200 pixels above the screen center. The social label—“self,” “Lin,” or “host”— was presented in Chinese (font size: 30), positioned 100 pixels below the shape. The target display appeared for 100 ms, after which a response prompt appeared instructing participants to “*Please make a response by pressing ‘y’ (correct match) or ‘n’ (incorrect match).*” Participants were instructed to respond as quickly and as accurately as possible. Any response latency exceeding 2,000 ms was recorded as “missed.” Following the response, a feedback screen showed “correct,” “incorrect,” or “missed” for 500 ms, based on the participant’s input.

Participants completed nine practice trials and were offered an additional set of nine trials if they felt more practice was needed. After the nine to 18 practice trials, the main experimental session began. This session consisted of 360 trials, during which each geometrical pattern was presented in 120 trials. Each pattern was presented with its corresponding social label for 60 trials (e.g., a circle was paired with “self” for participants whose correct pairing rule for a circle was “self”) and with each of the other two labels for 30 trials (e.g., a circle was paired with “Lin” or “host” for the same participants). The presentation order of the 360 trials was randomized. Participants were given a self-paced short break after every 120 trials. Throughout the task, the participants’ brain activity was recorded using an EEG device.

After completing the task, participants were asked to complete a questionnaire (see Supplementary Information: Appendix B) assessing dispositional empathy, situational empathy, and emotional response. All items were rated on a five-point Likert scale. Dispositional empathy was assessed by using the Interpersonal Reactivity Index (IRI) (Davis, 1983), which includes 28 items. Situational empathy was measured by using eight items adapted from Schutte and Stilinović (2017). Emotional responses were assessed with three items measuring positive valence, negative valence, and arousal, with questions including: “How strongly did this interview evoke positive emotions in you?” “How strongly did this interview evoke negative emotions in you?””How aroused did this interview make you feel?” For both the situational empathy and emotional response items, participants were instructed to reflect on their reactions toward Lin during the experimental task (shape–label matching task).

The entire experimental session lasted approximately 2 h, including EEG setup, the shape–label matching task, and time for participants to wash and dry their hair afterward. The shape–label matching task itself took approximately 30–40 min to complete. Each participant received 500 New Taiwan Dollars as compensation for their participation.

## Results

### Manipulation check

An analysis of variance (ANOVA) revealed that the ratings of situational empathy (Cronbach’s *α* = 0.93) differed significantly between the high-empathy (*M* = 3.60) and the low-empathy groups (*M* = 2.62) (*F* [1, 50] = 14.39, *p* <.001, *η*^*2*^_*p*_ =.22). Based on these results, our manipulation of empathy was successful.

### Potential confounding variables

We also assessed positive emotion, negative emotion, and arousal level as potential confounding variables. We first examined whether these variables differed between the high-empathy and low-empathy groups using analysis of variance (ANOVA). Interestingly, participants in the high-empathy group reported higher levels of positive valence (*M* = 2.88,* F* [1, 50] = 4.85, *p* =.03, *η*^*2*^_*p*_ =.09) than those in the low-empathy group (*M* = 2.23). No significant differences were observed for negative emotion (*F* [1, 50] = 0.02, *p* =.90, *η*^*2*^_*p*_ <.001) or arousal (*F* [1, 50] = 0.85, *p* =.36, *η*^*2*^_*p*_ =.02).

Readers may wonder why the high-empathy group reported a higher level of positive emotion than the low-empathy group after listening to a tragic story. Some participants noted that although the story about the university student Lin was tragic, they felt inspired by Lin’s bravery and resilience, which in turn elicited positive emotions. One possible way to address this potential confounding variable is to include it as a covariate in the subsequent analyses. However, given the significant correlation between positive valence and situational empathy (*r* = 0.38, *t*[50] = 2.87, *p* =.006), controlling for positive valence would also remove a significant portion of variance associated with situational empathy, which is the primary variable of interest in the present study. Therefore, we chose not to partial out the effect of positive valence. The potential confounding role of positive valence will be further discussed in the General Discussion.

Dispositional empathy (Cronbach’s *α* = 0.83) did not differ significantly between the two groups (*F* [1, 50] = 0.77, *p* =.39, *η*^*2*^_*p*_ =.02).

### Behavioral data: Sensitivity

Behavioral data were analyzed using the SDT. According to SDT, a higher value of *d'* indicates greater sensitivity to distinguishing between targets and non-targets. In this study, *d’*_*self*_*, d’*_*Lin*_* and d’*_*host*_ respectively represent participants’ sensitivity to shapes associated with “self,” “Lin,” and “host.” Specifically, *d’*_*self*_ refers to sensitivity to the difference between the self-associated shape (i.e., the geometrical shape associated with “self”) and the other shapes (those associated with “Lin” or “host”) when the label is “self.” Similarly, *d’*_*Lin*_ refers to the sensitivity to the difference between the Lin-associated shape and the other shapes when the label is “Lin,” and *d’*_*host*_ refers to the sensitivity to the difference between the host-associated shape and the other shapes when the label is “host.”

The calculation of *d’*_*self*_ is based on the hit rate and the false alarm rate to the self-associated shape as “signals.” The hit rate is defined as the proportion of correct “yes” responses in trials where the self-associated shape is paired with the “self” label. Conversely, the false-alarm rate indicates the proportion of incorrect “yes” responses in trials where either the Lin-associated or host-associated shape was paired with the “self” label. The following formula is used for this calculation:

$${{d}{\prime}}_{self}={\Phi }^{-1}\left(H\right)-{\Phi }^{-1}\left(F\right)$$

Here, Φ^−1^(H) and Φ^−1^(F) represent the inverse Gaussian functions, which convert the hit rate and the false alarm rate, respectively, into *z* scores. To prevent infinite values, which occur when hit rates or false alarm rates reach 100% or 0, we adjusted them according to an approach suggested by Stanislaw and Todorov (1999). Specifically, we utilized the formula (*n* – 0.5)/*n* or 0.5/*n*, where *n* indicated the number of signal or noise trials and was set to 60 in our study. The calculation of *d’*_*Lin*_ and *d’*_*host*_ followed a similar procedure, with the “signals” referring to trials with the Lin- and host-associated shapes, respectively.

**Fig. 2:**
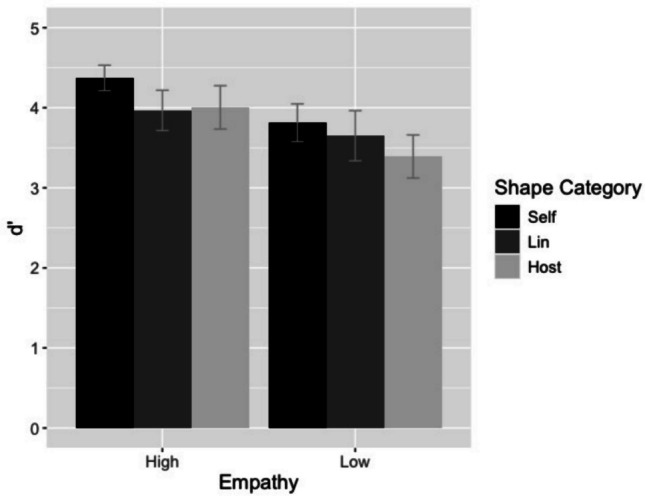
The mean *d’* values across experimental conditions. The error bars indicate 95% confidence intervals

We conducted an analysis of variance (ANOVA) on *d’* (Fig. 2), with empathy (high-empathy group versus low-empathy group) and shape category (self, Lin, and host) as independent variables. The results revealed a significant effect of empathy (*F* [1, 50] = 12.69, *p* =.001, *η*^*2*^_*p*_ =.20). There was also a significant effect of shape category (*F*[2, 100] = 9.99, *p* <.001, *η*^*2*^_*p*_ =.17). The *d’* for self-associated shape was significantly higher than the Lin-associated shape (*t*[51] = 3.10, *p* =.003) and the host-associated shape (*t*[51] = 4.40, *p* <.001), but the Lin-associated shape and the host-associated shape did not significantly differ (*t*[51] = 1.19, *p* =.24). The interaction did not reach statistical significance (*F* [2, 100] = 1.51, *p* =.23, *η*^*2*^_*p*_ =.03). We observed a self-prioritization effect in *d’*. In terms of empathy, the participants’ sensitivity values increased for all the shape categories, supporting the self–other distinction hypothesis.

### Behavioral data: Response bias

With the signal detection theory, we can also estimate the response bias index of *c.* The values of *c*_*self,*_* c*_*Lin*,_ and *c*_*host*,_ respectively, represent the response bias for self-associated shape, Lin-associated shape and host-associated shape. For example, *c*_*self*_ was calculated by using the following formula (Stanislaw & Todorov, 1999):

$$c=-\left({\Phi }^{-1}\left(H\right)+{\Phi }^{-1}\left(F\right)\right)/2$$

The terms Φ^−1^(H) and Φ^−1^(F) represent the inverse Gaussian functions that convert the hit rate and the false-alarm rate for shapes representing self into *z* scores. The calculations of *c*_*Lin*_ and *c*_*host*_ follow a similar process, with the “signals” referring to trials with Lin-associated and host-associated shapes. A higher *c* value indicates a more conservative response bias*.* For example, a higher *c*_*self*_ suggests a greater tendency to press “no” when viewing the label of “self.”

**Fig. 3:**
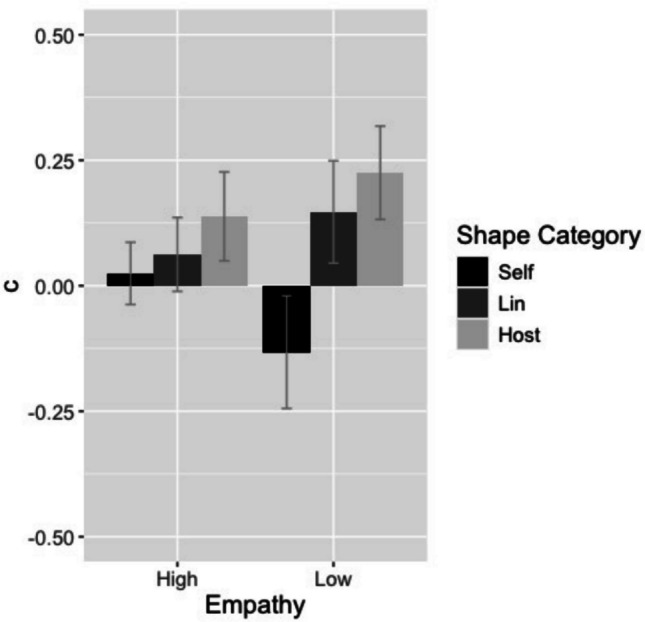
The mean *c* values across experimental conditions. The error bars indicate 95% confidence intervals

**Fig. 4:**
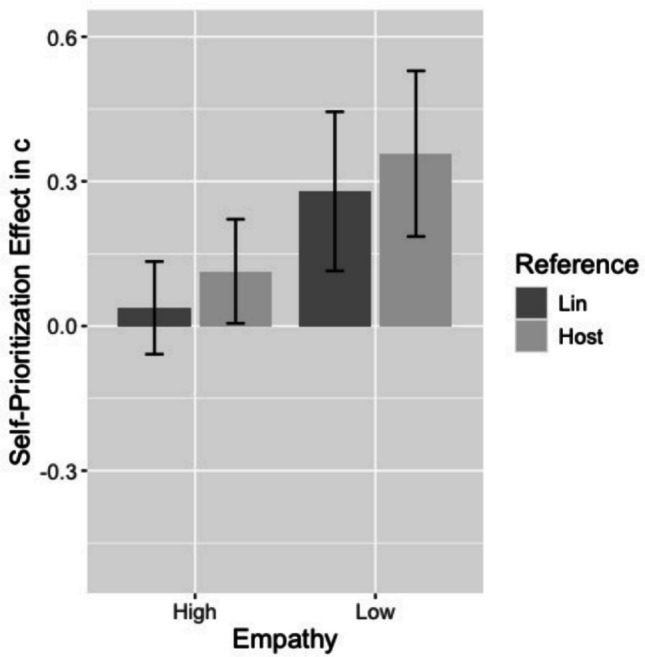
The self-prioritization effect using the Lin and host conditions as the references, estimated by lower *c* values (i.e., a higher tendency to press “yes”) in the self condition compared with the Lin or host condition

We conducted an analysis of variance (ANOVA) on *c* (Fig. 3), with shape category and empathy as independent variables. A significant interaction emerged (*F* [2, 100] = 4.75, *p* =.01, *η*^*2*^_*p*_ =.09). To further examine the interaction, we decomposed the effect of shape category into two components: the self-prioritization effect using Lin (the empathy target) as the reference and the self-prioritization effect using host (the irrelevant individual) as the reference. These effects were operationalized as the difference in *c* between the self condition and either the Lin or host condition, respectively (Fig. 4).

We then conducted a second ANOVA using self-prioritization effect as the dependent variable, with empathy and reference (Lin versus host) as factors. This analysis revealed a significant main effect of empathy (*F* [1, 50] = 7.77, *p* =.008, *η*^*2*^_*p*_ =.13), which did not significantly interact with reference (*F* [1, 50] = 0.001, *p* =.98, *η*^*2*^_*p*_ <.001). In other words, the magnitude of the self-prioritization effect on *c* was constant regardless of whether Lin or the host served as the reference. Importantly, this effect was reduced in the high-empathy group compared with the low-empathy group, consistent with the self–other merging hypothesis.

### Behavioral data: Reaction time

We also recorded the reaction time, following prior works using the shape-label matching paradigm (Sui et al., 2012, 2023). We included only trials with correct responses, which were then separated into matching and mismatching categories. Matching trials were those where the shape and the label aligned according to the instructed pairing rule (e.g., the self-associated shape with the label of “self”), whereas mismatching trials did not (e.g., the self-associated shape with the label of “Lin” or “host”).

We then conducted a three-way ANOVA using matching type, empathy, and shape category as factors. The full results are shown in Table 1. There was a significant interaction between matching type and shape category (*F* [2, 100] = 70.67, *p* <.001, *η*^*2*^_*p*_ =.59). For matching trials, the effect of shape category reached statistical significance (*F* [2, 102] = 56.74, *p* <.001, *η*^*2*^_*p*_ =.53). Pairwise comparisons showed that the self-associated shape led to shorter reaction times than the Lin-associated shape (*t*[51] = 7.37, *p* <.001) and the host-associated shape (*t*[51] = 9.07, *p* <.001). Lin-associated shape also led to shorter reaction time than the host-associated shape (*t*[51] = 2.64, *p* =.01). This replicated the study of Sui et al. (2023) as well as Amodeo et al. (2024). For the mismatching trials, shape category did not induce a significant effect (*F* [2, 102] = 0.98, *p* =.38, *η*^*2*^_*p*_ =.02).

**Table 1:** ANOVA results with reaction time as the dependent variable

	*df*	*F*	*p*	*η*^*2*^_*p*_
Empathy	1, 50	1.29	0.26	0.03
Shape category	2, 100	31.75	< 0.001	0.39
Matching type	1, 50	66.12	< 0.001	0.57
Empathy x matching type	1, 50	0.004	0.95	< 0.001
Empathy x shape category	2, 100	2.51	0.09	0.05
Matching type x shape category	2, 100	70.67	< 0.001	0.59
Empathy x matching type x shape category	2, 100	2.54	0.08	0.05

**Fig. 5:**
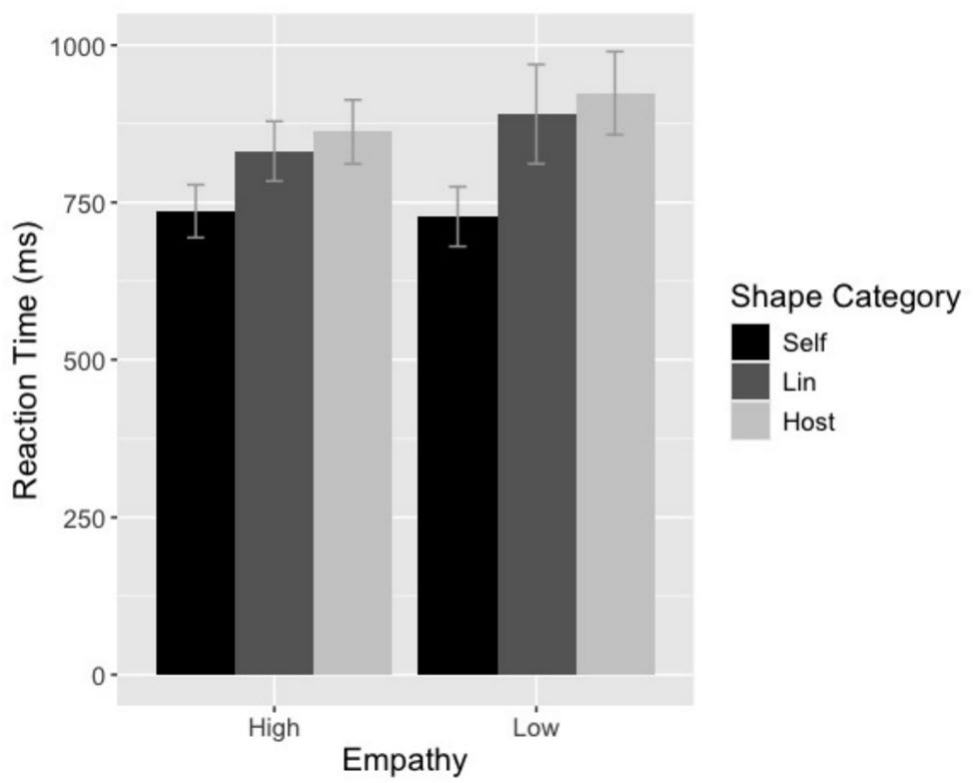
The mean reaction times (matching trials only) across experimental conditions. The error bars indicate 95% confidence intervals

There was a marginal three-way interaction among empathy, matching type and shape category (*F* [2, 100] = 2.54, *p* =.08, *η*^*2*^_*p*_ =.05). In matching trials (Fig. 5), shape category significantly interacted with empathy (*F* [2, 100] = 3.22, *p* =.04, *η*^*2*^_*p*_ =.06), whereas in mismatching trials, no such interaction was observed (*F* [2, 100] = 0.24, *p* =.79, *η*^*2*^_*p*_ =.005).

**Fig. 6:**
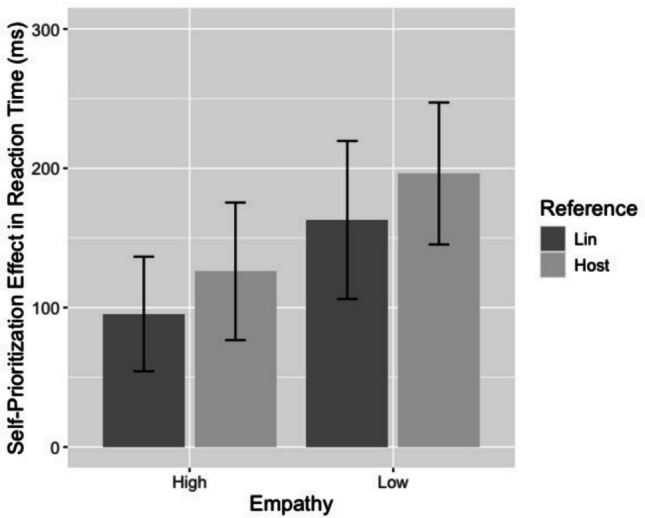
The self-prioritization effect in reaction time (matching trials only), estimated by the increase in reaction time from the self condition to the Lin or host condition

To further examine the significant interaction between shape category and empathy in matching trials, we decomposed the shape category effect into self-prioritization effects in reaction time, using Lin (RT_Lin_—RT_self_) and the host (RT_host_—RT_self_) as references (Fig. 6), analyzing only matching trials. ANOVA revealed a significant reduction in the self-prioritization effect for the high-empathy group than the low-empathy group (*F* [1, 50] = 4.62, *p* =.04, *η*^*2*^_*p*_ =.09), supporting the self–other merging hypothesis. Furthermore, the reduction in the self-prioritization effect was constant regardless of whether Lin or host was used as the reference, as indicated by the nonsignificant interaction between empathy and reference (*F* [1, 50] = 0.01, *p* =.91, *η*^*2*^_*p*_ <.001).

### EEG data: Preprocessing

The EEG data were recorded using Neuroscan SynAmps 2 (Compumedics Neuroscan Inc., Australia), with a sampling rate of 1,000 Hz. Data analysis was performed by using the EEGLAB toolbox (Delorme & Makeig, 2004) on MATLAB R2020b (MathWorks, Natick, MA). A 50 Hz low-pass filter and a 0.5 Hz high-pass filter were applied during preprocessing, followed by a down-sampling to 250 Hz. Subsequently, the data were visually inspected, and noisy segments were rejected. Independent component analysis (ICA) was used to correct EOG artifacts (Delorme et al., 2007). The continuous EEG data were then segmented into multiple epochs from − 200 to 1,000 ms relative to target onset. Baseline correction was applied using the 200-ms pretarget period.

### EEG data: N1

In Sui et al.(2023), the N1 component was measured at electrodes P5, P6, TPP7h, TPP8h, P7, and P8 within a 147–167 ms time window. In contrast, Amodeo et al. (2024) measured the N1 component at P7, PO7, P8, and PO8 between 190 and 280 ms. Because both studies included P7 and P8, we focused our analysis on these two sites. To determine our time window, we plotted the averaged waveforms at P7 and P8 and identified a clear peak negativity between 140 and 204 ms. We then identified the N1 amplitudes as the averaged amplitudes in this time window.

These N1 values were subjected to an ANOVA, with matching type, shape category, and empathy as independent variables. Channel (P7 versus P8) was also included as an additional factor. A significant interaction among channel, matching type, and shape category emerged (*F* [2, 100] = 11.26, *p* <.001, *η*^*2*^_*p*_ =.18). None of other main effects or interactions reached statistical significance (*ps* >.05). Consequently, we conducted separate analyses for P7 and P8.

**Fig. 7:**
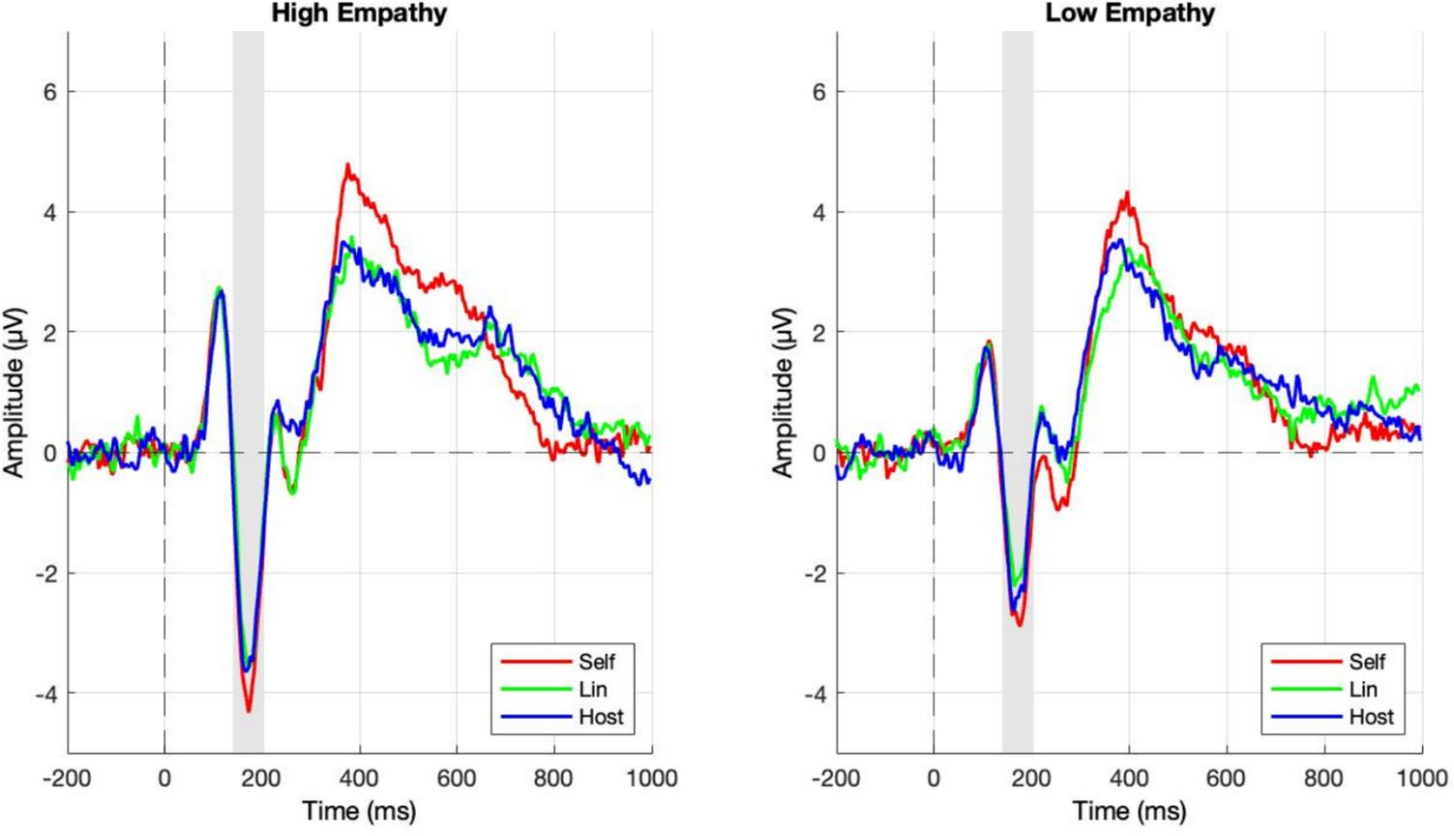
The averaged waveforms for the three shapes (associated with self, Lin, and host) in the high-empathy group (left panel) and the low-empathy group (right panel) for matching trials at P7. The grey areas indicate the time windows for the N1 component

For P7 (Fig. 7), a three-way ANOVA (Table 2) with matching type, shape category, and empathy as factors revealed a significant interaction between matching type and shape category (*F* [2, 100] = 6.75, *p* =.002, *η*^*2*^_*p*_ =.12). For matching trials, shape category had a significant main effect (*F* [2, 102] = 6.63, *p* =.002, *η*^*2*^_*p*_ =.12). Specifically, N1 amplitudes were more negative for the self-associated shape than for the Lin-associated shape (*t*[51] = 3.63, *p* =.001), marginally more negative for the self-associated shape than the host-associated shape (*t*[51] = 1.91, *p* =.06), and marginally more negative for the host-associated shape than the Lin-associated shape (*t*[51] = 1.71, *p* =.09). For mismatching trials, shape category had no significant effect (*F* [2, 102] = 1.00, *p* =.37, *η*^*2*^_*p*_ =.02).

**Table 2:** ANOVA results with N1 as the dependent variable at the P7 site

	*df*	*F*	*P*	*η*^*2*^_*p*_
Empathy	1, 50	1.04	0.31	0.02
Shape category	2, 100	0.99	0.38	0.02
Matching type	1, 50	0.17	0.68	.003
Empathy x matching type	1, 50	1.03	0.32	0.02
Empathy x shape category	2, 100	0.09	0.91	0.002
Matching type x shape category	2, 100	6.75	0.002	0.12
Empathy x matching type x shape category	2, 100	0.13	0.88	0.003

For P8, no significant main effect or interaction was observed (*ps* >.05).

These results at P7, located over the left hemisphere, demonstrated a self-prioritization effect, consistent with the findings of Sui et al. (2023) and Amodeo et al. (2024). In contrast, no significant effect was observed at P8, which is in the right hemisphere. However, the main variable of interest of the present study—empathy—did not significantly influence N1 amplitudes at either P7 or P8.

### EEG data: P3

**Fig. 8:**
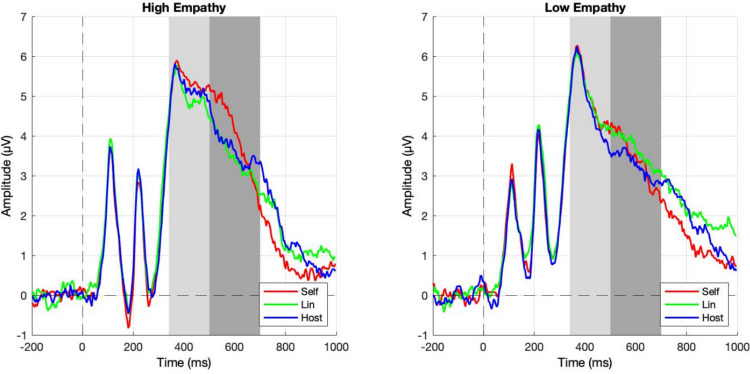
The averaged waveforms for the three shapes (associated with self, Lin, and host) in the high-empathy group (left panel) and the low-empathy group (right panel) at Pz. The light gray and dark gray areas indicate the time windows for the P3 and LPC components, respectively

In the study of Sui et al. (2023), the P3 component was indexed using Pz, PPO1h, PPO2h, and POz within the 340–500 ms time window. In the study by Amodeo et al. (2024), P3 was defined by the amplitudes from Pz, POz, P1, and P2 within a later time window of 500–700 ms. We focused on the Pz electrode, which was common to both studies. Averaged waveforms for correct-response trials in the high-empathy and low-empathy groups are shown in Fig. 8. To examine temporal dynamics, we first used the 340–500 ms time window to index P3, following the approach of Sui et al. (2023) and then applied the 500–700 ms window to capture the LPC, as in Amodeo et al. (2024).

The P3 amplitudes were analyzed using ANOVA (Table 3), with empathy, shape category and matching type as independent variables. A significant interaction between matching type and shape category emerged (*F* [2, 100] = 11.50, *p* <.001, *η*^*2*^_*p*_ =.19).

**Table 3:** ANOVA results with P3 as the dependent variable at the Pz site

	*df*	*F*	*p*	*η*^*2*^_*p*_
Empathy	1, 50	0.04	0.84	0.001
Shape category	2, 100	1.03	0.36	0.02
Matching type	1, 50	14.47	< 0.001	0.22
Empathy x matching type	1, 50	0.68	0.41	0.01
Empathy x shape category	2, 100	0.76	0.47	0.02
Matching type x shape category	2, 100	11.50	< 0.001	0.19
Empathy x matching type x shape category	2, 100	0.46	0.63	0.009

For matching trials, there was a significant main effect of shape category (*F* [2, 102] = 9.78, *p* <.001, *η*^*2*^_*p*_ =.16), driven by larger P3 amplitudes for the self-associated shape compared to the Lin-associated shape (*t*[51] = 3.39, *p* <.001) and the host-associated shape (*t*[51] = 3.88, *p* <.001), with no difference between the Lin-associated and the host-associated shapes (*t*[51] = 0.001, *p* >.99). For mismatching trials, there was also a significant main effect of shape category (*F* [2, 102] = 4.31, *p* =.02, *η*^*2*^_*p*_ =.08). Post-hoc comparisons showed smaller P3 amplitudes for the self-associated shape relative to both the Lin-associated (*t*[51] = 2.35, *p* =.02) and the host-associated shapes (*t*[51] = 2.47, *p* =.02), with no significant difference between the Lin-associated and the host-associated shapes (*t*[51] = 0.008, *p* =.99).

For matching trials, we observed a significant self-prioritization effect. However, the empathy manipulation did not exert any significant effects.

### EEG data: LPC

We then used the later time window, as in Amodeo et al. (2024), to index LPC. LPC amplitudes were analyzed using ANOVA (Table 4), with empathy, shape category, and matching type as independent variables.

**Table 4:** ANOVA results with LPC as the dependent variable at the Pz site

	*df*	*F*	*p*	*η*^*2*^_*p*_
Empathy	1, 50	0.06	0.81	0.001
Shape category	2, 100	1.50	0.23	0.03
Matching type	1, 50	26.53	< 0.001	0.35
Empathy x matching type	1, 50	0.16	0.69	0.003
Empathy x shape category	2, 100	3.16	0.047	0.06
Matching type x shape category	2, 100	5.58	0.005	0.10
Empathy x matching type x shape category	2, 100	0.19	0.82	0.004

Again, a significant interaction between shape category and matching type emerged (*F* [2, 100] = 5.58, *p* =.005, *η*^*2*^_*p*_ =.10). For matching trials, the main effect of shape category was significant (*F* [2, 102] = 5.07, *p* =.008, *η*^*2*^_*p*_ =.09): The self-associated shape elicited significantly larger LPC amplitudes than the Lin-associated shape (*t*[51] = 3.21, *p* =.002) and the host-associated shape (*t*[51] = 2.03, *p* =.047), with no significant difference between the Lin-associated and host-associated shapes (t[51] = 0.68, *p* =.50). These findings reveal a self-prioritization effect. For mismatching trials, the effect of shape category was not significant (*F* [2, 102] = 2.38, *p* =.10, *η*^*2*^_*p*_ =.05).

**Fig. 9:**
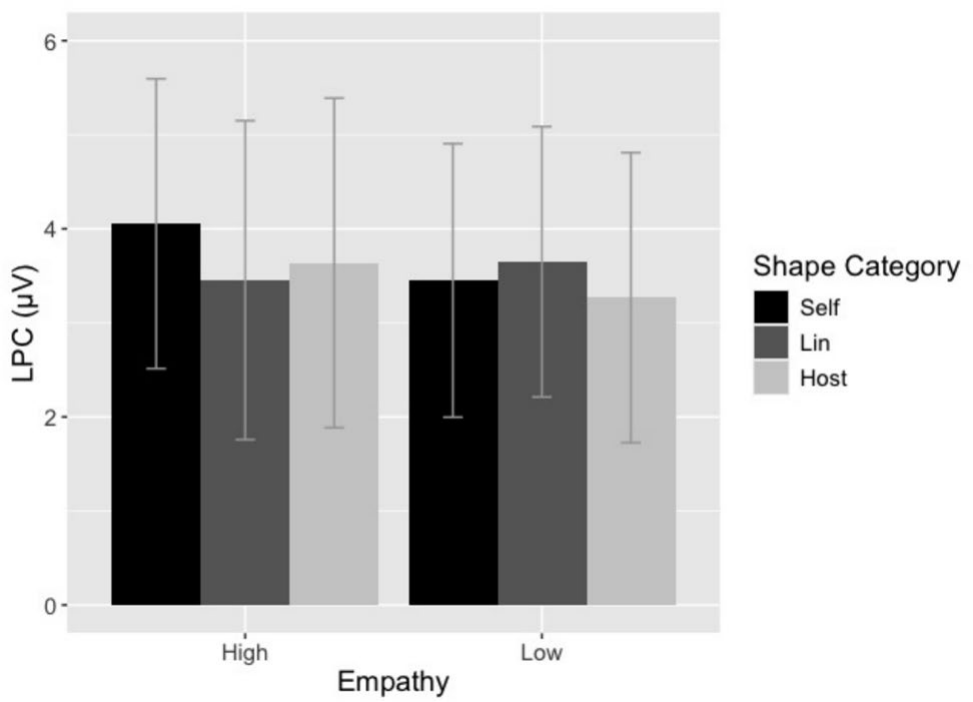
The mean LPC amplitude across experimental conditions. The error bars indicate 95% confidence intervals

**Fig. 10:**
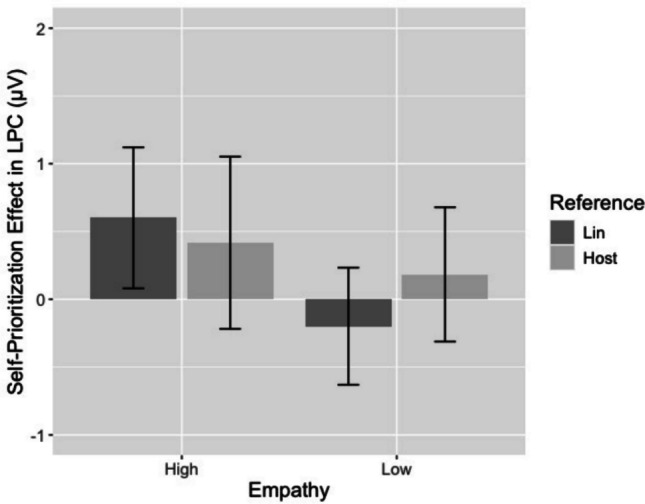
The self-prioritization effect, estimated by the decrease in LPC amplitude from the self condition to either the Lin or the host condition

We also observed a significant interaction between shape category and empathy (*F* [2, 100] = 3.16, *p* =.047, *η*^*2*^_*p*_ =.06). Please refer to Fig. 9 for the mean LPC amplitudes across conditions. We further examined this significant interaction by decomposing the shape category effect into the self-prioritization effect in LPC using Lin (LPC_self_ – LPC_Lin_) and host (LPC_self_ – LPC_host_) as references (Fig. 10). The ANOVA showed a significant interaction between reference and empathy (*F* [1, 50] = 4.58, *p* =.04, *η*^*2*^_*p*_ =.08). The self-prioritization effect was only significant when Lin was used as a reference (*F* [1, 50] = 6.15, *p* =.02, *η*^*2*^_*p*_ =.11). In contrast, no significant effect was found when the host was used as the reference (*F* [1, 50] = 0.36, *p* =.55, *η*^*2*^_*p*_ =.007).

**Fig. 11:**
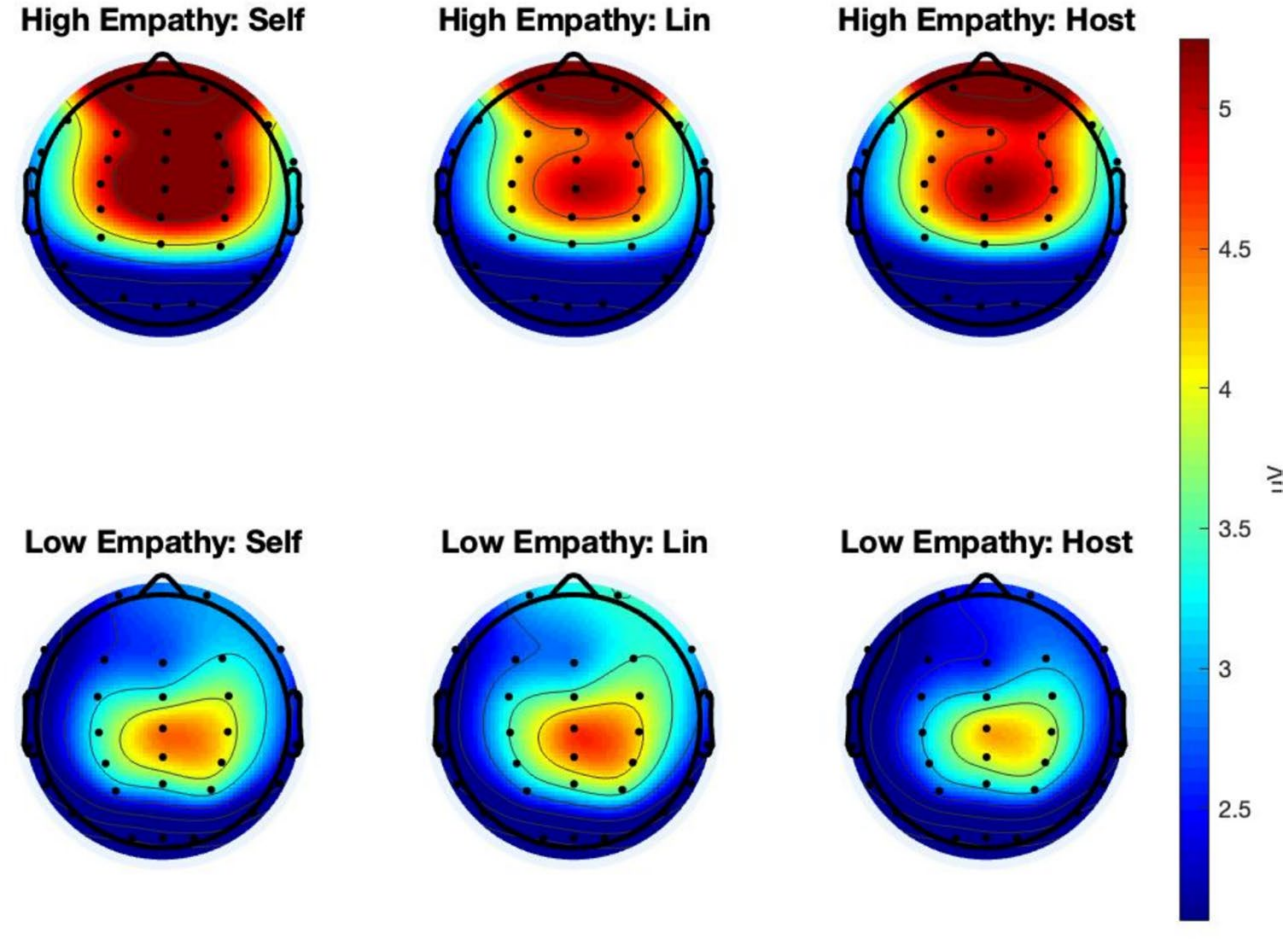
EEG topographies of the averaged potentials in the 500–700 ms time window across conditions

EEG topographies for the averaged potentials in the 500–700 ms time window across conditions are shown in Fig. 11.

## Discussion

### Summary of results

In the present study, we aimed to test whether empathy leads to self–other merging or self–other distinction. Participants were instructed to associate three geometric shapes with three different social labels and to perform a shape–label matching task. Different indices of measurement revealed different patterns. The sensitivity index *d’* showed an overall increase from the low- to high-empathy group. The response bias index *c* exhibited a significantly lower level of self-prioritization in the high-empathy group than in the low-empathy group. Reaction time revealed a similar trend to response bias, with marginal statistical significance. For the LPC obtained from EEG recordings, self-prioritization was greater in the high-empathy group than in the low-empathy group. Overall, the *d’* and LPC results suggest a self–other distinction effect, whereas the reaction time and *c* results indicate a self–other merging effect.

### Self–other distinction: d’

The sensitivity index *d’* reflected perceptual-level processing characteristics (Correa et al., 2005; Green & Swets, 1966). In this study, *d’* was generally higher for the high-empathy group than the low-empathy group. More specifically, participants in the high-empathy group were more sensitive to the differences among different shape categories, suggesting that empathizing with someone may enhance the perceived distinctiveness among individuals. One might argue that *d’* only revealed a main effect of empathy, whereas the self–other distinction hypothesis would predict an interaction between empathy and shape category. However, the calculation of *d’* involved both targets (signals) and nontargets (noise). For instance, computing *d’*_*self*_ requires estimating the hit rate from trials with the self-associated shape and the false-alarm rates from trials with Lin- and host-associated shapes. Similarly, calculating *d’*_*Lin*_ requires using performance from trials with the self-associated shape to estimate the false alarm rate. If empathy enhances the distinctiveness between self-related information and nonself-related information, it could reduce false alarms for *d’*_*Lin*_, thereby increasing its value. This may explain the lack of a significant interaction involving *d’*, because a biased enhancement of self-related processing could elevate both *d’*_*Lin*_ and *d’*_*host*_, diminishing the effect size of the interaction.

### Self–other distinction: LPC

The P3 component has been traditionally associated with the allocation of attentional resources (Polich, 2007; Polich & Kok, 1995). A common method for eliciting a P3 response is the oddball paradigm, in which a low-probability target item (the oddball) is embedded among frequent nontarget items. The rarer the oddball, the higher the resulting P3 amplitudes (Polich & Margala, 1997), reflecting the detection of ecologically significant events. Recent studies suggest that the P3 component may also indicate self-representation (Liu et al., 2019; Niu et al., 2020), likely mediated by attention. Self-relevant stimuli, such as rare events (or oddballs), tend to convey personally significant messages and thus automatically capture attention. In our study, the P3 amplitudes were higher for the self-associated shape, replicating the studies of Sui et al. (2023). However, the effect of empathy emerged slightly later (500–700 ms), typically referred to as the LPC. This suggests that self-prioritization may occur earlier in the processing stream than empathy-related effects.

While the LPC has more commonly been associated with memory paradigms, it can also reflect attentional processing. For example, Kissler et al. (2009) found enhanced LPC responses (450–650 ms) for the word class (adjectives or nouns) that participants were instructed to attend to, indicating an attentional effect. Given that our paradigm did not vary the memory load of different shape categories, we interpret the LPC effect here as reflecting attentional processes. A notable effect in our data was that empathy-induced self–other distinction emerged only when the Lin-associated shape served as the reference, but not when the host-associated shape did. This suggests that, in the context of self–other distinction, the term “other” may specifically refer to the empathy target (i.e., Lin) rather than to unrelated individuals or objects. Thus, empathy may accentuate the distinction between the self and the empathy target, producing a target-selective effect.

To integrate these findings, we propose a potential model of empathy’s perceptual influence: Self-relevant stimuli attract early attention, producing a self-prioritization effect as early as 147–167 ms (as reflected in the N1 component) and extending through the P3 window. The empathy-related effect appeared slightly later in the LPC time window and may serve to amplify the distinction between self-related information and information related to the empathy target. This amplification could contribute to higher *d’* values for the self-associated shape. Because *d’* values for Lin- and the host-associated shapes are also calculated by using performance on self-shape trials, a preferential enhancement of the self-associated shape could also elevate *d’* for Lin- and host-associated shapes.

### Lateralization of N1

One surprising finding is the lateralization of the N1 component. In our analysis, the self-prioritization effect on N1 was only significant at site P7 (left hemisphere) but not P8 (right hemisphere). Such left-lateralized N1 effects have been reported in prior studies involving linguistic stimuli (Bentin et al., 1999; Spironelli & Angrilli, 2009; Van Setten et al., 2016). Although the shapes in our study were nonverbal and visually simple, they were arbitrarily associated with social labels, which might have endowed them with symbolic or semantic value. We speculated that this symbolic encoding of shape–label association may have engaged left-hemisphere processes more strongly, resulting in left-lateralized N1 effect.

### Self–other merging: c and reaction time

The *c* can be interpreted as a decision criterion; that is, the threshold for making a “yes” response. In this study, participants adopted a more conservative criterion in affirming a match when presented with the Lin- or host-associated shapes compared to the self-associated shape. However, this difference was smaller in the high-empathy group, suggesting that the criteria applied to the self, Lin- and host-associated shapes became more similar.

One might argue that because only Lin was the empathy target, any criterion shift should occur only for the Lin-associated shape, but not for the host-associated shape. Our interpretation is as follows: As shown in Fig. 3, the effect in our study appears to be driven primarily by a criterion change for the self-associated shape. It is possible that the apparent self–other merging was driven more by reduced attachment to the self (Wu et al., 2010) rather than increased alignment specifically with the empathy target. If empathy primarily reduces attachment to the self, then it would not necessarily produce differences between the empathy target and an unrelated individual. Instead, it would lead to a global reduction in self-related processing. Nevertheless, this explanation is post-hoc. Future research is needed to examine the degree to which empathy can produce truly target-selective effects.

A similar pattern emerged in reaction times, with empathy reducing the latency differences across responses to the self-, Lin-, and host-associated shapes. Compared with other indices (*d’, c,* and various ERP components), reaction times (RT) reflect a broader range of processing levels. Variations in RT can arise from perceptual, attentional, decisional, and response-related processes. In our data, the effect of empathy on RT was driven by its interaction with matching type and shape category, although this interaction was only marginally significant (*p* =.08). It is possible that empathy exerted opposing influences at different stages of processing, resulting in a net effect that was attenuated. Although the interaction did not reach conventional significance, we conducted follow-up simple main effect analysis and observed a pattern more consistent with self–other merging during matching trials. Importantly, RT alone does not allow us to determine the specific level it reflects. However, given that the RT pattern more closely resembled response bias (*c*) than perceptual sensitivity (*d’*), we are inclined to interpret the empathy-related RT effect as being more influenced by high-level decisional factors than by early perceptual processing.

Together, these findings provide a nuanced view of empathy. Empathy appears to increase perceptual and attentional sensitivity to differences between self and other, while simultaneously encouraging a more equitable response threshold across these distinctions. In other words, empathy sharpens perceptual awareness of label differences while reducing response bias favoring the self.

### Empathy: Neural and cognitive mechanisms

In the self-to-other model proposed by Bird and Viding (2014), exposure to another person’s emotional state triggers a similar emotional state in the observer, a process known as emotional contagion. For empathy to occur, however, individuals must go beyond mere contagion and attribute the experienced emotion to the other person. Decety and Lamm (2006) similarly argued that empathy involves both emotional contagion and self–other awareness.

Emotional contagion is thought to be supported by shared neural circuits between self and other, such as the anterior cingulate cortex, or ACC (Singer et al., 2004). In contrast, self–other awareness involves recognizing whether an emotion or sensation belongs to oneself or another. Accumulating evidence points to the right inferior parietal cortex as a key region for comparing internal and external signals (Blakemore & Frith, 2003; Lamm et al., 2007). For instance, hemodynamic responses in the right inferior parietal lobe were greater when individuals imagined how others feel than when they imagined the feelings for themselves (Lamm et al., 2007).

The two components of empathy, emotional contagion and other-tagging, may account for self–other merging and self–other distinction, respectively. Perhaps, emotional contagion tends to exert more influence when the task involves high-level judgement or categorization. For example, in the study by Batson et al. (1997), emotional contagion in high-empathy participants may have led them to classify themselves as more similar to others, as reflected in the choice of more overlapping pairs on the Inclusion of Others Scale (Aron et al., 1992). Similarly, Riečanský et al. (2020) found that empathy-related neural activity was positively associated with the extent to which participants classified another person’s body part as part of their own. Chen et al. (2020) showed that neural responses to others’ pain (an indicator of empathy) were positively correlated with individuals’ self-rated interdependent self-construal, that is, the extent to which people emphasize social relationships and group memberships.

In contrast, for perceptual or attentional tasks, emotional contagion may play a less prominent role, allowing other-tagging to shape self–other distinction. For instance, in studies by Mattan et al. (2016) and Chiu and Yeh (2018), participants were required to determine other people’s visual perspectives. Individuals with higher levels of empathy were better at differentiating self-related from other-related information.

One insight from the present study is that “self” might be a multilevel construct. At lower levels, the self refers to any sensory stimuli related to oneself, whereas at higher levels, it reflects an abstract concept of I-ness. When empathy operates at a low level, the other-tagging component may enhance the distinctiveness of self-related stimuli. In contrast, at high levels, the emotional contagion component may blur the abstract boundary between the self and others, leading individuals to perceive others as part of themselves.

### Limitations and future directions

The potential limitations of the present study can be discussed from two main perspectives: the manipulation of empathy and the measurement of self-representation.

A key concern with our empathy manipulation lies in its potential confounding with positive valence. Participants in the high-empathy group reported higher levels of positive valence than those in the low-empathy group. Thus, the observed effects—particularly those associated with empathy—might have been driven by positive valence rather than empathy per se. While we cannot completely rule out this possibility empirically, we are more inclined to interpret our findings as effects of empathy rather than positive valence.

Our manipulation caused self–other distinction at the perceptual and attentional level, and self–other merging at the response level. Two possible mechanisms could account for the observed effects: empathy and positive valence. Prior research has demonstrated empathy’s influence on self–other distinction (Chiu & Yeh, 2018; De Guzman et al., 2016; Decety & Lamm, 2006; Krol & Bartz, 2022; Mattan et al., 2016) and on self–other merging (Banissy & Ward, 2007; Batson et al., 1997; Chen et al., 2020; Riečanský et al., 2020; Seiryte & Rusconi, 2015; Wu et al., 2010). Based on this body of work, we attribute our effects more confidently to empathy.

In contrast, there is limited support for positive valence as a mechanism underlying self–other merging. To our knowledge, only one study has demonstrated its effect on self–other merging (Waugh & Fredrickson, 2006). However, the measure of “positive emotion” used in this study included an item assessing sympathy; therefore, it is likely that the reported “positive” emotion in that study was confounded with empathy, further supporting our interpretation that empathy, rather than positive valence, was the primary driver of the observed effects.

Regarding the measurement of self, the issue is inherently complex, as the concept of “self” is itself multifaceted. Across languages, words referring to “I” or “me” are ubiquitous, suggesting that self-representation is a fundamental and universal feature of human cognition. However, defining and measuring the self scientifically is challenging due to the lack of a well-established operational definition. In the present study, we used participants’ performance on the shape–label matching task as an indicator of self-representation. This paradigm has been widely used to demonstrate the self-prioritization effect in prior studies (Dalmaso et al., 2019; Desebrock et al., 2018; Lo, 2021; Macrae et al., 2018; Martínez-Pérez et al., 2024; Sui et al., 2012, 2013; 2015), supporting its reliability.

Nonetheless, the question of validity remains: Does the self-prioritization effect in the shape–label matching task truly reflect self-representation? This effect can be observed across various paradigms. For example, Amodeo et al. (2021) demonstrated the self-prioritization effect using a shape–label matching task, a trait adjectives task, and a visual search task; yet the strength of the relationships among self-prioritization effects varied across tasks. They suggested that self might be heterogeneous and multifaceted. Similarly, Nijhof et al. (2020) reported a lack of correlation between self- prioritization effect in the shape–label matching task and a task involving attention.

We speculated that different paradigms may probe different levels or aspects of self-representation. Even within the same paradigm, different measures may tap into distinct processes. Notably, both Nijhof et al. (2020) and Amodeo et al. (2021) defined self-prioritization effect in the shape–label matching task based on reaction time, which reflects a broad range of cognitive processes. Nijhof et al. (2020) also included overall accuracy, which may also conflate multiple stages of processing. Had alternative measures been used, different correlation patterns might have emerged. Future research should systematically compare measures across paradigms to clarify the validity of self-representation assessments.

One possible future direction is to identify EEG signatures of self–other merging. In the present study, we used the behavioral index of *d’* and the ERP recording of the LPC to demonstrate a self–other distinction effect. However, we did not identify any ERP component reflecting self–other merging. One potential candidate for this purpose is the lateralized readiness potential, which is associated with response activation processes and is measured as the difference in signal between channels contralateral and ipsilateral to the response side (Gratton et al., 1988). Unfortunately, the task design in the present study did not involve lateralized responses, preventing us from measuring the lateralized readiness potential. Future studies using lateralized responses could help explore this possibility.

## Conclusion

Using the shape–label matching task, we found evidence that empathy can be associated with both self–other distinction and self–other merging. Perceptual sensitivity and attentional indices suggested that empathy may enhance the differentiation between self and others, whereas response bias and reaction time patterns suggested that empathy may blur the boundaries between them. These findings highlight that both empathy and self-representations are multifaceted, and their interaction is likely to involve multiple mechanisms operating at different levels. Our study provides one possible account of how these processes may relate, but further research is necessary to clarify the conditions under which empathy promotes distinction versus merging and to uncover the underlying mechanisms in more detail.

## Supplementary Information

Below is the link to the electronic supplementary material.

Supplementary file1 (DOCX 21 KB)

## Data Availability

The program code used for data analysis is available from the corresponding author upon reasonable request.

## Declarations

### Open practices statements

The data and materials for the experiments reported in this manuscript are not publicly available due to restrictions imposed by the ethics committee of the corresponding author’s institution but may be available from the corresponding author upon reasonable request and with permission of the ethics committee. None of the experiments was preregistered.
