# Supplementary material for: Distinct effects of empathy on self–other processing revealed by different behavioral and EEG indices
Source: Cogn Affect Behav Neurosci. 2026 Mar 4;26(4):1817–34. doi: 10.3758/s13415-026-01416-2 (PMC13385067; doi:10.3758/s13415-026-01416-2)
Supplement: Supplementary file 1 — Supplementary file1 (DOCX 21 KB) [file 13415_2026_1416_MOESM1_ESM.docx]

# **Appendix A**

Interview Transcript Used in the Experiment

**Host**: Mr. (Ms.) Lin is a university student, who has tragically lost his (her) parents in a car accident recently. They have no insurance, and Lin must now take care of his (her) 11-year-old brother and eight-year-old sister. Mr. (Ms.) Lin, thank you so much for agreeing to this interview today. I know your family has gone through such a tragic trauma recently. You’ve received a lot of help from people, thanks to the media reports and charitable organizations. Can you talk with us about how this has affected you and what plans you have for the future?

**Lin**: To me, this feels like a nightmare! I still can’t believe that my parents are gone. First of all, I am very grateful for the help from so many kind people, which has helped me and my siblings get by. But the road ahead is long. We can’t rely on other people’s help forever. So, the most important thing for me now is to graduate as soon as possible, and find a job to support my family.

**Host**: You’re still in the university, aren’t you? Taking care of your brother and sister while studying is quite challenging. How do you manage your time?

**Lin**: My brother and sister still go to school during the week. When I don’t have class, I work part-time at a gas station or a restaurant. After school, I pick them up, make dinner, help them with their homework, and put them to bed. After they fall asleep, I do my own work. In my free time at night, I also take on some home-based jobs for extra income so that we don’t have to rely on others’ help as much.

**Host**: It sounds like you have a lot on your plate, balancing your studies and taking care of your siblings. Have you thought about seeking help from others?

**Lin**: I have thought about it. But my parents didn’t have siblings, and my grandparents are no longer around. I’m hesitant to ask distant relatives for help because they don’t know our situation well. It would feel strange to suddenly reach out to them. So, I do as much as I can myself. My siblings have lost their parents, and I am their pillar of support. They are my responsibility. Sometimes, it does feel like 24 hours in a day aren’t enough, but looking at it another way, our days are very fulfilling.

**Host**: What about after you graduate? Do you have any plans? Your siblings are still young, and there will be ongoing expenses for their education and living costs.

**Lin**: For now, I plan to see if there’s anything valuable at home that we don’t need and sell it to get some money for my siblings’ future education. After graduation, I’ll work on stabilizing my income to ensure they are well taken care of. My brother did mention that when he starts junior high school, he’ll work part-time to help me to cover some expenses and take care of our younger sister. But I told him to focus on his studies and not worry about money for now. I think our parents would support this decision.

**Host**: It seems like you’re always thinking about your family and your siblings. Have you considered what you truly want to do?

**Lin**: I did think about it when my parents were still around. I wanted to become a pilot after graduation. Flying to different countries is one of my dreams. But now, I’m the only one who can take care of the family, so I’ll think about it after my siblings are grown up.

**Host**: From what I know, pilots earn a good salary, which could be a stable source of income. Have you considered this?

**Lin**: The salary is indeed good, but being away for long periods makes it difficult to take care of my siblings personally. I want to spend more time with them as they grow up. It might be exhausting, but I believe that if my parents knew, they would be relieved and happy to see that I’ve grown up and can take care of my siblings. That’s enough for me.

**Host**: It must be really tough for you. Your parents would be very proud. Thank you so much for sharing your story with us today. Thank you!

# **Appendix B**

Questionnaire Used in the Experiment

(All the items were rated on a 5-point Likert scale)

| **Interpersonal Reactivity Index** | 1. I daydream and fantasize, with some regularity, about things that might happen to me. |
| --- | --- |
|  | 2. I often have tender, concerned feelings for people less fortunate than me. |
|  | 3. I sometimes find it difficult to see things from the “other guy's” point of view (*reverse coded*). |
|  | 4. Sometimes I don't feel very sorry for other people when they are having problems (*reverse coded*). |
|  | 5. I really get involved with the feelings of the characters in a novel. |
|  | 6. In emergency situations, I feel apprehensive and ill-at-ease. |
|  | 7. I am usually objective when I watch a movie or play, and I don't often get completely caught up in it (*reverse coded*). |
|  | 8. I try to look at everybody's side of a disagreement before I make a decision. |
|  | 9. When I see someone being taken advantage of, I feel kind of protective towards them. |
|  | 10. I sometimes feel helpless when I am in the middle of a very emotional situation. |
|  | 11. I sometimes try to understand my friends better by imagining how things look from their perspective. |
|  | 12. Becoming extremely involved in a good book or movie is somewhat rare for me (*reverse coded*). |
|  | 13. When I see someone get hurt, I tend to remain calm (*reverse coded*). |
|  | 14. Other people's misfortunes do not usually disturb me a great deal (*reverse coded*). |
|  | 15. If I'm sure I'm right about something, I don't waste much time listening to other people's arguments (*reverse coded*). |
|  | 16. After seeing a play or movie, I have felt as though I were one of the characters. |
|  | 17. Being in a tense emotional situation scares me. |
|  | 18. When I see someone being treated unfairly, I sometimes don't feel very much pity for them (*reverse coded*). |
|  | 19. I am usually pretty effective in dealing with emergencies (*reverse coded*). |
|  | 20. I am often quite touched by things that I see happen. |
|  | 21. I believe that there are two sides to every question and try to look at them both. |
|  | 22. I would describe myself as a pretty soft-hearted person. |
|  | 23. When I watch a good movie, I can very easily put myself in the place of a leading character. |
|  | 24. I tend to lose control during emergencies. |
|  | 25. When I'm upset at someone, I usually try to "put myself in his shoes" for a while. |
|  | 26. When I am reading an interesting story or novel, I imagine how I would feel if the events in the story were happening to me. |
|  | 27. When I see someone who badly needs help in an emergency, I go to pieces. |
|  | 28. Before criticizing somebody, I try to imagine how I would feel if I were in their place. |
| **Situational empathy** | 1. I imagined myself to be in Lin's situation |
|  | 2. I felt as if I were in Lin's shoes. |
|  | 3. I felt touched by Lin's situation. |
|  | 4. I felt sorry for Lin. |
|  | 5. I tried to see things from Lin's point of view. |
|  | 6. I felt compassion for Lin. |
|  | 7. I tried to take the perspective of Lin. |
|  | 8. I felt protective towards Lin. |
| **Emotion** | 1. How strongly did this interview evoke positive emotions in you? |
|  | 2. How strongly did this interview evoke negative emotions in you? |
|  | 3. How aroused did this interview make you feel? |
